# How Auxin and Cytokinin Phytohormones Modulate Root Microbe Interactions

**DOI:** 10.3389/fpls.2016.01240

**Published:** 2016-08-18

**Authors:** Stéphane Boivin, Camille Fonouni-Farde, Florian Frugier

**Affiliations:** Institute of Plant Sciences – Paris Saclay, Centre National de la Recherche Scientifique, Institut National de la Recherche Agronomique, Université Paris-Sud, Université Paris Diderot, Université d’Evry, Université Paris-SaclayGif-sur-Yvette, France

**Keywords:** auxin, cytokinin, pathogens, endomycorrhiza, ectomycorrhiza, *Rhizobium*, root nitrogen fixing symbiosis, legume nodulation

## Abstract

A large range of microorganisms can associate with plants, resulting in neutral, friendly or hostile interactions. The ability of plants to recognize compatible and incompatible microorganisms and to limit or promote their colonization is therefore crucial for their survival. Elaborated communication networks determine the degree of association between the host plant and the invading microorganism. Central to these regulations of plant microbe interactions, phytohormones modulate microorganism plant associations and coordinate cellular and metabolic responses associated to the progression of microorganisms across different plant tissues. We review here hormonal regulations, focusing on auxin and cytokinin phytohormones, involved in the interactions between plant roots and soil microorganisms, including bacterial and fungi associations, either beneficial (symbiotic) or detrimental (pathogenic). The aim is to highlight similarities and differences in cytokinin/auxin functions amongst various compatible versus incompatible associations.

## Introduction

Plant–microorganism interactions have received more and more attention due to the benefits they confer to crop productivity by improving nutrient uptake, increasing plant growth and conferring biotic and abiotic stress tolerance (Yang et al., 2009; de Zelicourt et al., 2013; Grover et al., 2013). Identifying communication systems and signals that determine the beneficial or detrimental outcomes of plant–microorganism interactions is a key to improve defense responses without decreasing beneficial (e.g., symbiotic) associations.

**FIGURE 1:**
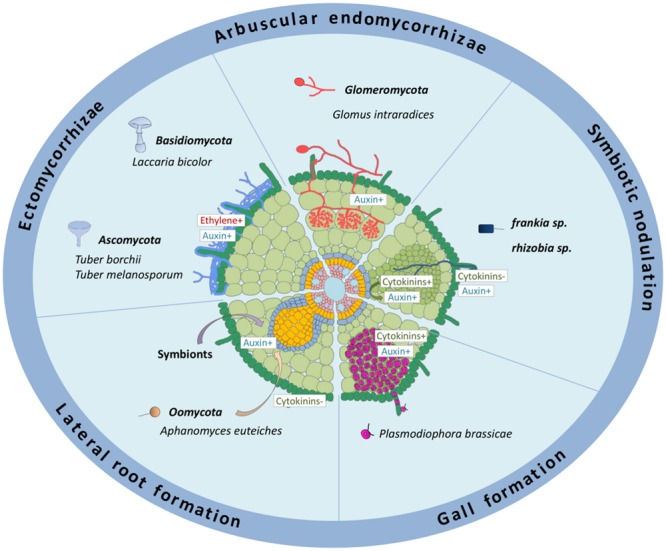
**Roles of auxins and cytokinins in different biotic interactions affecting the root system.** The schematized root represents different interactions occurring on root systems from different plant species: ectomycorrhizae, formed mainly on tree roots; arbuscular endomycorrhyzae, occurring in most land plants; symbiotic nodulation in legumes (≪rhizobia≫) or actinorhizal plants (≪*Frankia*≫); galls formed in roots of some *Brassicaceae* plants. The effect of symbionts and oomycete pathogens on lateral root development is also depicted. Auxin metabolism and/or response is positively associated to ECM, AM, rhizobia and *Frankia* symbiotic interactions (either in relation to infections and/or to nodule organogenesis), to the interaction with several root pathogens (acting either positively or negatively on pathogenic susceptibility), and positively associated to root gall formation in response to *P. brassicae*. Auxins produced by different rhizospheric microorganisms stimulate lateral root formation. Cytokinin metabolism and/or response is associated to rhizobia nodulation, either positively in the cortex or negatively in the epidermis, to the interaction with several root pathogens (acting mostly positively on pathogenic susceptibility), and positively associated to root gall formation in response to *P. brassicae.* +, indicates a positive effect; -, a negative effect. Dark green: root hairs and epidermis; pale green, cortex; middle green, nodule primordium; blue, endodermis; yellow, pericycle; pink, stele tissues including vascular bundles.

Different symbiotic associations with plant roots exist, either with fungi or bacteria (**Figure 1**). These symbioses are mutualistic, leading to reciprocal exchanges between fungi or bacterial microorganisms and host plants: soil nutrients or fixed atmospheric nitrogen versus carbon skeletons generated through photosynthesis (Udvardi and Poole, 2013; Schweiger and Müller, 2015). These interactions can be established between ectomycorrhizal (ECM) fungi from the *Basidiomycota* and *Ascomycota* phyla and many forest trees (Anderson and Cairney, 2007; Diagne et al., 2013; Raudaskoski and Kothe, 2015), arbuscular endomycorrhizal (AM) fungi from the *Glomeromycota* phylum with most of land plants (Schüβler et al., 2001; Smith and Read, 2010; Foo et al., 2013; Gutjahr and Parniske, 2013), and nitrogen-fixing bacteria such as *Rhizobium* sp. and *Frankia* sp. with specific species belonging to the Rosid family, leading to the formation of new root lateral organs called nodules (Dénarié et al., 1992; Franssen et al., 1992; Soltis et al., 1995; Perret et al., 2000; Santi et al., 2013; Svistoonoff et al., 2014) (**Figure 1**). Unlike AM fungi and N_2_-fixing bacteria, ECM fungi do not enter inside plant host root tissues and cells. A mycelial mantle is formed by the fungi around short lateral roots and develops between root epidermal and cortical cells, to form a highly branched structure, called the Hartig net (Peterson and Massicotte, 2004; Anderson and Cairney, 2007; Raudaskoski and Kothe, 2015) (**Figure 1**).

In all symbiotic interactions, the recognition of microorg-anisms and plant roots as symbiotic partners is the first critical step to allow a tight beneficial metabolic association (Bonfante and Requena, 2011; Gough and Cullimore, 2011; Geurts et al., 2012; Singh and Parniske, 2012; Genre et al., 2013; Raudaskoski and Kothe, 2015). In the case of nitrogen fixing symbioses, the formation of a new organ, the root nodule, is additionally essential to generate microaerobic conditions allowing differentiated bacteria to fix atmospheric nitrogen (e.g., production of leghemoglobin proteins that chelate oxygen, preventing inhibition of the bacterial nitrogenase enzyme ensuring nitrogen fixation; Ferguson et al., 2010; Desbrosses and Stougaard, 2011; Oldroyd, 2013) (**Figure 1**). Exchanges of molecular signals, including on the one hand flavonoids and phytohormones produced by legume plants, and on the other hand Myccorhization Factors or Nodulation Factors (NFs) respectively secreted by AM fungi or rhizobia, are required for the recognition of symbiotic partners. In addition, these signaling pathways participate in decreasing root defense responses, preparing root tissues for infection by the symbiont, and in the case of nitrogen fixing bacteria, initiating root cortical cell divisions leading to nodule organogenesis (Oldroyd et al., 2011; Oldroyd, 2013; Gourion et al., 2015) (**Figure 1**).

Foiling plant defenses is therefore critical for a successful host infection, either for symbiotic partners or pathogens (Robert-Seilaniantz et al., 2011; Pieterse et al., 2012; De Vleesschauwer et al., 2014). To penetrate, colonize and hijack nutrients from host plants, pathogenic bacteria and fungi that infect roots have developed different strategies, including the modification of phytohormonal responses to their advantage. Some pathogens are able to directly synthesize phytohormones affecting plant growth and development, forming new sinks in which nutrients are easily accessible (e.g., *Agrobacterium tumefaciens* galls, or *Plasmodiophora brassicae* clubroot galls; **Figure 1**). Therefore, keeping the control over hormonal pools and signaling pathways is crucial for host plants to both establish beneficial microorganism interactions and prevent pathogenic invasions.

### Auxin and Cytokinin Regulations in Plant – Fungus Symbioses

The two types of plant-fungal symbioses, AM and ECM, display different hormonal regulation requirements, notably regarding auxins and cytokinins. A production of cytokinins (isopentenyl and *cis*-zeatin) by ECM fungi was identified (Morrison et al., 2015). In addition, ECM fungi, such as *Laccaria bicolor, Tuber borchii* and *T. melanosporum*, produce measurable amounts of auxin (indole-3-acetic acid; IAA), resulting in morphological changes of host roots (*Cistus incanus*) either depending on a direct (contact) or indirect (diffusible signal) interaction with the fungus (Karabaghli et al., 1998; Felten et al., 2009; Splivallo et al., 2009). Accordingly, the presence of fungal mycelia reduces root growth and increases root branching of the host plant, as observed in *C. incanus* and *Populus trichocarpa*, and similarly as an exogenous auxin treatment does (Felten et al., 2009; Splivallo et al., 2009). Interestingly, in *Arabidopsis thaliana*, the ectomycorrhizal truﬄe mycelium also promotes root hair growth as well as lateral root formation, indicating that these may not be symbiosis-specific traits (Splivallo et al., 2009). In addition to the production of auxins, *L. bicolor* is able to release ethylene which activates the plant auxin synthesis pathway (Splivallo et al., 2009). The production of these two hormones is required to promote root hair growth to an equivalent level as the truﬄe mycelium does. Altogether, these observations suggest that ethylene production by the ECM fungi may induce auxin production in the host plant, therefore reinforcing the effect of direct auxin production on root development and notably on the promotion of lateral root formation which will then be infected by new mycelia (**Figure 1**). The local activation of auxin responses in the first tier of root tip columella cells of poplar and *Arabidopsis* in response to an indirect contact with *L. bicolor* has been documented using the auxin-response reporter construct *DR5::GFP*, and this activation was inhibited by a Polar Auxin Transport inhibitor (PAT; Splivallo et al., 2009). In addition, transcriptomic analyses in poplar roots inoculated with the ECM fungus *L. bicolor* revealed an increased expression of auxin-related genes such as members of the *GH3* (*Gretchen Hagen3*) gene family involved in auxin conjugation, as well as of *P. trichocarpa PtaPIN4* and *PtaPIN9* auxin eﬄux carriers essential for PAT (Felten et al., 2009). Interestingly, *L. bicolor* inoculation induces lateral root formation in wild-type *A. thaliana* but not in the *pin2* mutant (AtPIN2 is the closest Arabidopsis relative of PtaPIN9 in poplar). This result is consistent with the essential role of PAT in controlling lateral root development induced by the presence of the symbiotic ECM fungus (Felten et al., 2009).

In contrast to the ECM symbioses, no comprehensive change was observed in auxin levels in *Tropaeolum majus* upon inoculation with different AM strains (Jentschel et al., 2007). Nevertheless, a role of auxins in the AM symbiosis was proposed notably in relation to the stimulating effect of the AM inoculation on lateral root formation (Fusconi, 2014). Indeed, several mutants affected in auxin-related developmental responses, such as the *Pisum sativum bushy* mutant that displays a lower IAA concentration in shoots and roots and the tomato (*Solanum lycopersicum*) *diageotropica* auxin-resistant and *polycotyledon* hyperactive PAT mutants show a reduced mycorrhizal colonization (Hanlon and Coenen, 2011; Foo, 2013) (**Figure 1**). However, the expression of the strigolactone (SL) biosynthetic *PsCCD8* (Carotenoid Cleavage Dioxygenases 8) gene is decreased in the *bushy* mutant (Foo et al., 2013), suggesting that the auxin effect on AM symbiosis may be at least partly due to a decrease in the SL biosynthesis. Several auxin-responsive genes were identified as induced in AM roots, such as a specific *GH3* tomato gene expressed in cells colonized by fungi (Liao et al., 2015). As the symbiotic expression of this marker could be disconnected from its auxin-induction, this suggests that an AM signaling GH3-related response may have evolved at least partially independently of auxin signaling. The expression of the *DR5* auxin response reporter was additionally detected in *S. lycopersicum, Medicago truncatula*, and *Oryza sativa* root cells containing arbuscules (Etemadi et al., 2014). Finally, although no analysis of the AM colonization capacity of mutants directly affected in auxin perception or polar transport is yet available, overexpression of a microRNA (miR393) that indirectly downregulates the expression of auxin receptor genes (i.e., *Transport Inhibitor Response1* and *Auxin-related F-Box* genes) led to the formation of underdeveloped arbuscules in *S. lycopersicum, M. truncatula*, and *O. sativa* roots, suggesting that hampering auxin perception in arbuscule-containing cells negatively affects their formation (Etemadi et al., 2014).

Cytokinins were also proposed to be involved in the AM symbiosis since an increase of cytokinin levels in leaves and roots was detected in AM infected plants (Allen et al., 1980). However, it remains unclear if the cytokinins were produced by the host plant or by the AM fungus (Allen et al., 1980; Barker and Tagu, 2000; Shaul-Keinan et al., 2002). No AM phenotype was detected in the *M. truncatula cre1* (*cytokinin response 1*) mutant defective in a cytokinin receptor essential for symbiotic nodulation (Plet et al., 2011; Laffont et al., 2015), suggesting that at least the CRE1-dependent cytokinin signaling is not essential for the AM symbiotic interaction.

### Auxin and Cytokinin Regulations of Nitrogen-Fixing Root Nodule Symbioses

**FIGURE 2:**
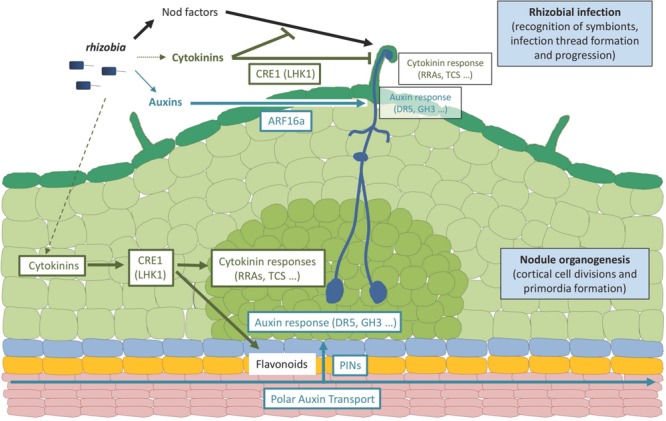
**Roles of auxins and cytokinins in legume-rhizobia symbiotic interactions.** The scheme represents an early rhizobial infection event in a legume (*Medicago truncatula*) root leading to the formation of an indeterminate nodule. Rhizobia produce specific Nod factor signaling molecules as well as auxin and cytokinin hormones. A major contribution of Nod factor signals in establishing rhizobial infection events (i.e., symbiont recognition, infection thread formation and progression) in root hairs has been demonstrated, whereas cytokinins produced by rhizobia may only have a minor contribution, and a role for a WT-level bacterial production of auxins remains to be established. Plant and/or bacterial hormones lead to the activation of cytokinin and auxin responses in infected epidermal cells, respectively, monitored by Type A RRs (RRA) genes or the TCS (Two Component output Sensor) cytokinin-response marker and the GH3 (Gretchen Hagen3) auxin responsive gene or the DR5 auxin-response marker. The activation of cytokinin responses in the root epidermis may lead to an inhibition of Nod factor signaling and/or rhizobial infections whereas the activation of auxin responses may promote rhizobial infections. The activation of these root epidermal cytokinin responses depends on the CRE1 (Cytokinin Response 1) receptor (LHK1, standing for Lotus Histidine Kinase1, being its functional homolog in *Lotus japonicus*) whereas the activation of the auxin epidermal responses depends on ARF16a (Auxin Response Factor 16a, a transcription factor). A cytokinin response is additionally activated in dividing cortical cells and nodule primordia, also depending on the CRE1 (LHK1) receptor, promoting nodule organogenesis even in the absence of symbiotic signals (rhizobia or Nod factors). An activation of auxin responses is observed in the cortex downstream of the CRE1-cytokinin pathway, involving local changes in polar auxin transport associated to the activation of specific flavonoid metabolite production and changes in PIN (PIN-formed) auxin-eﬄux carriers expression and accumulation. Ultimately, the activation of these auxin and cytokinin responses is linked to modulations of the cell cycle leading either to infection thread formation (epidermis) or cell divisions (cortex). It remains open if the auxin and/or cytokinin hormonal pools may, in addition to originate from plants and/or bacteria, accumulate upon rhizobial/Nod factors activation specifically in the infected epidermis, in the activated cortex, or in both root tissues. In addition, divergences in these hormonal responses are observed between determinate and indeterminate nodule types (e.g., *L. japonicus* versus *M. truncatula* model legumes), notably regarding the role of CRE1 versus LHK1 cytokinin receptors in regulating infections in the epidermis, the involvement of polar auxin transport in root inner tissues, and the contribution of a local auxin biosynthesis in these different root tissues. Dark green: root hairs and epidermis; pale green, cortex; middle green, nodule primordium; blue, endodermis; yellow, pericycle; pink, stele tissues including vascular bundles.

Several studies have highlighted the involvement of auxin and cytokinin phytohormones in the regulation of the *Rhizobium* nitrogen-fixing symbiotic interaction. Allen et al. (1953) showed that an exogenous application of a PAT inhibitor could induce the formation of nodule-like structures on alfalfa roots, in the absence of *Rhizobium*. However, the structure of these organs can be considered as more similar to roots than to legume nodules. The inhibition of PAT was found to also induce pseudonodule formation in *M. truncatula* roots (Rightmyer and Long, 2011), further indicating that nodule organogenesis involved a local auxin accumulation. It was later shown that combined auxin and cytokinin exogenous treatment on pea roots led to cortical cell divisions, which occur at the onset of nodule organogenesis (Libbenga et al., 1973) (**Figure 1**). The positive effect of cytokinins in the initiation of nodule organogenesis was additionally documented in different legumes where exogenous applications of cytokinins induce cortical cell divisions, amyloplast accumulation, and the expression of early nodulation markers (early nodulins; Torrey, 1961; Dehio and de Bruijn, 1992; Bauer et al., 1996; Jiménez-Zurdo et al., 2000; Mathesius et al., 2000; Murray et al., 2007; Tirichine et al., 2007). In addition, the ectopic expression of a CytoKinin oXidase/deshydrogenase gene from *A. thaliana* (*AtCKX3*), involved in the degradation of the cytokinin bioactive pool, or the downregulation of either a cytokinin activation gene from *M. truncatula* (MtLOG1, standing for LOnely Guy 1) or an Iso-PentenylTransferase biosynthetic gene from *L. japonicus* (*LjIPT3*), lead to a reduced nodule organogenesis (Lohar et al., 2004; Chen Y. et al., 2014; Mortier et al., 2014), suggesting that endogenous cytokinins act positively on nodulation. However, a reduced nodulation is also observed when the *MtLOG1* gene is overexpressed (Mortier et al., 2014) and in a *L. japonicus* mutant affecting the NF-induced *CKX3* gene (Reid et al., 2016), indicating that a tight regulation of cytokinin levels is required and/or that a negative symbiotic function of cytokinins exists. As rhizobia can secrete bioactive auxins (Camerini et al., 2008; Bianco and Defez, 2010) and cytokinins (Phillips and Torrey, 1972; Sturtevant and Taller, 1989), it was proposed that these two hormones could contribute to the induction of nodule formation, in addition to other bacterial symbiotic signals such as NFs. Indeed, a *Rhizobium nod^-^* strain, unable to synthesize NFs and form nodules but genetically modified to secrete the *trans*-zeatin cytokinin, is able to induce the formation of nodule-like structures expressing nodulation markers (Cooper and Long, 1994). The secretion of cytokinins by wild-type rhizobia does not seem, however, essential for nodulation, even though it might have a minor contribution (Kisiala et al., 2013; Podlešáková et al., 2013). In agreement, van Zeijl et al. (2015) showed that cytokinins are accumulated in wild-type roots in the absence of *Rhizobium* following a 3 h NF treatment. This suggests that the cytokinin accumulation required for nodulation mainly originates from the host plant. Concerning auxins, the over-production of this phytohormone in *Rhizobium* positively regulates nodulation and nodule meristem size (Camerini et al., 2008), and auxin-response reporter *DR5* and/or *GH3* fusions revealed that rhizobia or NFs can locally inhibit PAT and induce a local auxin accumulation in dividing cortical cells and nodule primordia in *M. truncatula, L. japonicus, T. repens*, and *Vicia sativa* (Mathesius et al., 1998; Boot et al., 1999; Pacios-Bras et al., 2003; Breakspear et al., 2014; Ng et al., 2015) (**Figure 2**). Interestingly, the accumulation of auxin (Indole-3-Acetic Acid, IAA) in *Rhizobium* inoculated plants was found to be dependent on cytokinin signaling pathways (Ng et al., 2015).

The involvement of cytokinin signaling pathways in the regulation of nodulation was first highlighted using a RNAi approach specifically targeting different putative cytokinin receptors in *M. truncatula* roots, revealing that only the silencing of *MtCRE1* led to cytokinin insensitive roots which developed a reduced number of nodules (Gonzalez-Rizzo et al., 2006) (**Figure 2**). Similarly, the *lhk1/hit1* (*lotus histidine kinase 1/hyperinfected 1*) mutant of *L. japonicus*, affecting the closest homolog of MtCRE1, showed a strongly reduced nodulation capacity associated with a hyperinfection phenotype (Murray et al., 2007). Strikingly, the *L. japonicus snf2* (*spontaneous nodule formed 2*) mutant carrying a gain of function mutation in the LHK1 cytokinin receptor led to the formation of “spontaneous nodules” in the absence of *Rhizobium* (Tirichine et al., 2007) (**Figure 2**). Altogether, these results unambiguously indicate that cytokinins and the LHK1/CRE1 pathway play a positive role in nodulation by inducing cortical cell divisions and nodule organogenesis (**Figures 1** and **2**). The fact that the *lhk1* and *cre1* mutants still form some nodules strongly suggest that a functional redundancy exists within the cytokinin receptor CHK family. Accordingly, mutants affecting other cytokinin receptors than *cre1/lhk1* also have reduced nodulation efficiencies, even though to a lesser extent (Held et al., 2014; Boivin et al., 2016). In addition, these reduced nodulation phenotypes are stronger when LHK1 or CRE1 is affected, further indicating a predominant role for CRE1/LHK1 in nodule initiation.

As previously mentioned, compatible rhizobia can locally inhibit auxin accumulation and/or PAT. Accordingly, in *L. japonicus*, the induction of an auxin response is observed both in *Rhizobium*-induced nodules and in *snf2* spontaneous nodules (Suzaki et al., 2012, 2013), indicating that this response is associated at least with early nodule organogenesis, downstream of the LHK1 cytokinin signaling pathway (**Figure 2**). The inhibitory effect of a *Rhizobium* application on PAT in *M. truncatula* roots is not observed in the *cre1* mutant, who displays an increased PAT capacity and accumulate excessively polarly localized MtPINs auxin eﬄux carriers (Plet et al., 2011) (**Figure 2**). Recently, Ng et al. (2015) showed that the nodulation defect of the *cre1* mutant could be partially complemented by an auxin transport inhibitor, as well as by specific flavonoids able to inhibit PAT (such as naringenin, isoliquiritigenin, and kaempferol; **Figure 2**). Overall, these data highlight that the activation of auxin responses and flavonoid accumulation downstream of cytokinins and LHK1/CRE1 is tightly associated with dividing cortical cells and nodule primordia formation.

Type B Response Regulators (RRBs; Heyl et al., 2013) are transcription factors directly regulating cytokinin primary response genes such as the *Nodulation Signaling Pathway 2* (*NSP2*) gene that encodes a GRAS-type transcription factor essential for early nodulation (Kaló et al., 2005), directly linking NF and cytokinin signaling pathways (Ariel et al., 2012). Other direct cytokinin signaling targets are associated to negative feedback regulations, such as the *Cytokinin Oxidase/deshydrogenase 1* (*CKX1*) gene involved in cytokinin degradation and type A Response Regulators (RRAs) thought to act as negative regulators of cytokinin signaling. Several *RRA* genes, used as markers of the activation of cytokinin responses, are associated with symbiotic nodulation in *M. truncatula*, such as *MtRRA4* that is expressed in dividing root cortical cells, nodule primordia and meristems, (Plet et al., 2011) (**Figure 2**) and *MtRRA9* and *MtRRA11* that are upregulated both in the root epidermis and in the root cortex in response to rhizobia or NFs (Op den Camp et al., 2011) (**Figure 2**). An RNAi construct targeting *MtRRA9* as well as *MtRRA4, MtRRA5*, and *MtRRA11* significantly reduces nodule formation in *M. truncatula*, suggesting a positive role of RRAs in nodulation (Op den Camp et al., 2011). As observed when affecting the cytokinin pool, the downregulation of the type A RR *MtRRA9* reduces nodulation, suggesting a negative function of cytokinins in nodulation (Op den Camp et al., 2011). Different versions of the “Two Component output Sensor” (TCS) reporter, used as a proxy to monitor the activation of the cytokinin primary response, revealed either an expression only in nodule primordia (TCS reporter, van Zeijl et al., 2015), in contrast to *MtRRA9* and *MtRRA11* (Op den Camp et al., 2011), or first in the epidermis and then in the cortex (TCSn reporter, Jardineau et al., 2016) (**Figure 2**). In *L. japonicus*, the TCS reporter expression was detected both in the infected root epidermis and in cortical cells during nodule organogenesis (Held et al., 2014), consistent with the induction of *LjRRA4, LjRRA6*, and *LjRRA8* genes in response to a NF treatment (Op den Camp et al., 2011), but following a different kinetic, being first expressed in the root cortex and then in the epidermis. The expression of several cytokinin biosynthesis and signaling genes, including *CRE1* and *MtRRA2, MtRRA8, MtRRA9* and *MtRRA10*, is rapidly upregulated by NFs or *Rhizobium* in the *M. truncatula* root epidermis and/or in isolated root hairs (Breakspear et al., 2014; Liu et al., 2015; Jardineau et al., 2016) (**Figure 2**). Accordingly, Pro*_LHK1_*:GUS and Pro*_CRE1_*: GUS fusions were detected in response to *Rhizobium* both in the root epidermis and in dividing cortical cells (Held et al., 2014; Boivin et al., 2016; Jardineau et al., 2016). This epidermal expression correlates with the *Rhizobium* hyperinfected phenotype observed in the *hit1/lhk1* mutant, suggesting that in addition to a positive role in nodule organogenesis, a negative function of cytokinins regulating epidermal infections may exist (Murray et al., 2007; Held et al., 2014) (**Figures 1** and **2**). Interestingly, in *M. truncatula*, the cytokinin/CRE1 pathway negatively regulates the NF-induction of the *ENOD11* epidermal infection marker (**Figure 2**), and reducing the cytokinin pool specifically in the epidermis positively impacts nodulation (Jardineau et al., 2016) (**Figure 1**).

Several auxin-related genes, such as members of the *Auxin Response Factor* (*ARF16a*), *GH3* (*GH3.1*), *Indole-3-Acetic Acid* (*IAA9*) and *Small Auxin Up RNA* (*SAUR1*) families, are also induced in the root epidermis and/or root hairs by *Rhizobium* and/or NFs (Breakspear et al., 2014; Jardineau et al., 2016) (**Figure 2**). In agreement, the auxin-response reporter *DR5* fusion was detected in *Rhizobium* infected root hairs as well as the auxin-responsive *GH3.1* gene, suggesting a role for auxins in epidermal infections (Breakspear et al., 2014; Laplaze et al., 2015) (**Figures 1** and **2**). Accordingly, an *arf16a* mutant shows a reduced rhizobial infection efficiency even though the number of nodule primordia and differentiated nodules remains unchanged, suggesting a positive role of auxins in the earliest stages of the rhizobial infection. A local accumulation of auxins depending on the AUX1 influx carrier was linked to *Frankia* nitrogen-fixing bacterial infections in the *Casuarina glauca* actinorhizal host plant forming symbiotic nodules evolutionary related to the legume nodulation (Péret et al., 2007; Perrine-Walker et al., 2010). Using a dominant-negative version of an auxin signaling repressor gene expressed in actinorhizal nodules, Indole-3-Acetic Acid7 (IAA7), to specifically inhibit auxin signaling in *Frankia*-infected cells, an increased actinorhizal nodulation was observed (Champion et al., 2015). This suggests a model where auxins, notably produced by *Frankia* symbiotic bacteria, induce the degradation of IAA7 and thus activate auxin-signaling, ultimately leading to an inhibitory negative feedback on nodulation. Finally, an activation of flavonoid pathways was also detected in the root epidermis and in root hairs in response to rhizobia and/or NFs (Breakspear et al., 2014; Chen et al., 2015; Jardineau et al., 2016), pointing to potential connections with cytokinin and auxin responses as recently described in early nodule organogenesis (Ng et al., 2015). Overall, these data indicate that in addition to their roles in nodule organogenesis, auxins, cytokinins and flavonoids may also regulate symbiotic bacterial infection (**Figures 1** and **2**).

Cytokinins and potentially auxins additionally likely regulate later symbiotic nodulation stages such as the nitrogen fixation metabolism, as suggested by transcriptomic analyses of laser-dissected differentiated nodule zones (Roux et al., 2014). Concerning auxins, it was recently reported that an auxin-overexpressing *S. meliloti* free-living strain showed transcriptome changes reminiscent of a differentiated nitrogen-fixing bacteroid, suggesting that auxins may affect bacteria differentiation depending on an unknown signaling pathway (Defez et al., 2016). Changes in auxin metabolism and response occurring in a wild-type *Rhizobium* strain during its differentiation within nodules however remain to be demonstrated. Concerning cytokinins, the downregulation of the *Iso-PentenylTransferase 3* (*LjIPT3*) gene in *L. japonicus* differentiated nodules, decreases nodule nitrogenase activity (Chen Y. et al., 2014). Recent studies, respectively using a *ckx3* mutant or *cre1* and related *chk* cytokinin receptor mutants, indicate that cytokinins affect nitrogen fixation efficiency both in *L. japonicus* and in *M. truncatula* (Boivin et al., 2016; Reid et al., 2016). Interestingly, a genomic clone corresponding to the closest CHK cytokinin receptor from the *Arabidopsis* non–symbiotic plant, AHK4/CRE1, is able to rescue early *cre1* nodulation defects (i.e., the number of nodules per plants) but not to complement the nitrogen fixation deficiency (Boivin et al., 2016). Noteworthy, three *L. japonicus* hemoglobin encoding genes (*LjGLB161, LjGLB2* and *LjGLB3-1*) are transcriptionally regulated by cytokinins (Bustos-Sanmamed et al., 2011), and in *O. sativa*, the expression of some hemoglobin genes may be directly regulated by RRB cytokinin signaling transcription factors (Ross et al., 2004). This may imply that a potential direct connection between cytokinins and the nitrogen fixation metabolism exists. Finally, it was recently proposed that cytokinins could be involved in a systemic shoot-to-root negative regulation of nodulation (Sasaki et al., 2014). More detailed analyses are therefore required to understand and integrate the various roles of auxins and cytokinins at these different symbiotic nodulation stages.

### Auxin and Cytokinin Regulations in Plant–Pathogen Interactions

In the past decades, most of the studies on plant pathogen interactions have focused on how the pathogens infect plant aerial organs (e.g., leaves). Auxins and cytokinins have been recently highlighted to act in defense responses either depending on other defense-related hormones such as Salicylic Acid and Jasmonic Acid, or independently (reviewed in Naseem and Dandekar, 2012). It should be noted that the function of auxins and cytokinins in defense and immunity responses largely depends on the plant and organ involved, probably because of their divergent developmental roles notably in shoots versus roots. It is only recently that data have been gained using root pathosystems (Chen Y.C. et al., 2014). Some root pathogens are able to synthesize auxin- and cytokinin-like molecules (Estruch et al., 1991; Argueso et al., 2009), indicating that the production of these two hormones is not restricted to either beneficial (symbiotic) or detrimental (pathogenic) microorganisms. Amongst the best described examples, *Agrobacterium tumefaciens* and *A. rhizogenes* are soil pathogenic bacteria targeting dicot plants (Smith and Townsend, 1907; Costantino et al., 1980). These bacteria carry a plasmid containing a Transfer-DNA (T-DNA) region that can be integrated into the plant genome (Liu and Kado, 1979; Kutáček and Rovenská, 1991; Lee et al., 2009). The *A. tumefaciens* T-DNA encodes two transcripts, named *iaaH* and *iaaM*, encoding auxin biosynthetic enzymes (Wood et al., 2001), and the *trans*-*zeatin synthesizing* (*tzs*) gene involved in cytokinin biosynthesis (Akiyoshi et al., 1984, 1987; Hwang et al., 2010). The integration of genes encoding these phytohormonal biosynthetic enzymes into the host plant genome leads to cell proliferation and a gall formation in the case of *A. tumefaciens*, or to root organogenesis in the case of *A. rhizogenes* “hairy roots”. Another well studied root pathogen is *P. brassicae*, which causes the clubroot disease in cruciferous plants such as *Brassica napus* (rapeseed) and *A. thaliana* (Hwang et al., 2012). The clubroot disease is characterized by the formation of galls on infected roots (**Figure 1**), leading to plant premature senescence. A microarray transcriptomic analysis performed on infected *A. thaliana* plants versus non-infected plants identified amongst differentially expressed genes, phytohormone-associated genes such as members of the auxin-related *GH3* gene family, or genes involved in the cytokinin biosynthesis (*AtIPT3* and *AtIPT8*), cytokinin degradation (*AtCKX1* and *AtCKX6*), cytokinin perception (*AHK4/CRE1*) and cytokinin signaling (ARR5 and ARR10; Siemens et al., 2006). Cytokinins and auxins were additionally functionally associated with early steps of the *P. brassicae* – *Arabidopsis* interaction in relation to the re-initiation of cortical cell divisions to form root galls, since an accumulation of isopentenyladenine and an enhanced auxin and cytokinin-related gene expression were identified (Ando et al., 2006; Devos et al., 2006; Schuller et al., 2014) (**Figure 1**). Interestingly, a link with flavonoid metabolic pathways was additionally highlighted since an accumulation of three types of flavonoids (naringenin, kaempferol and quercetin) was detected during clubroot gall formation (Päsold et al., 2010). Strikingly, an endophytic fungus, *Heteroconium chaetospira*, was described as a competitor for root cortical cell colonization, suppressing clubroot disease in rapeseed, and upregulating an auxin biosynthesis gene (*BnAAO1* for Ascorbic Acid Oxidase; Lahlali et al., 2014).

Amongst well-described plant pathogens infecting the root system, *Aphanomyces euteiches* is an oomycete causing strong damage to legume crops (Gaulin et al., 2007). Infected roots become brown and necrotic, leading to a reduction in water and nutrient uptake, and later, to leaf chlorosis and plant death (Gaulin et al., 2007). Interestingly, the resistance against this root pathogen is correlated with an increased capacity of the host plant to form lateral roots (Djébali et al., 2009) (**Figure 1**). The *M. truncatula* mutant affecting the MtCRE1 cytokinin receptor show an increased rate of survival in response to the pathogen, and this tolerance is correlated with the higher ability of this mutant to form lateral roots (Laffont et al., 2015), a developmental phenotype also observed in *Arabidopsis* cytokinin receptor mutants (Chang et al., 2013). In addition, a high-density Genome Wide Association Study (GWAS) revealed that a locus linked to *Aphanomyces* tolerance was potentially encoding an IPT cytokinin biosynthetic enzyme (Bonhomme et al., 2014), further suggesting the involvement of cytokinins in plant-pathogenic interactions (**Figure 1**).

*Fusarium oxysporum* is an ascomycete fungus belonging to a broad group containing non-pathogenic as well as pathogenic species. Root pathogenic strains are able to infect a wide range of plants including cotton, tomato, banana and *Arabidopsis* (Chen Y.C. et al., 2014). A microarray transcriptomic analysis performed in cotton infected roots (Dowd et al., 2004) and a RNAseq analysis performed on *F. oxysporum* infected *Arabidopsis* root tissues revealed changes in auxin-related gene expression, such as members of the *GH3, PIN, IAA* and *ARF* gene families (Lyons et al., 2015). Both *in vitro* and *in vivo* exogenous applications of auxins improve tomato root growth but also prevent *F. oxysporum* spore germination, suggesting a positive role of auxins in the plant resistance to *F. oxysporum* (Sharaf and Farrag, 2004).

Finally, one of the most famous and destructive soil-borne bacteria is *Ralstonia solanacearum*, causing a rapid vascular wilt disease to more than 200 species, including legumes, tomato, potato, tobacco, banana, and *Arabidopsis* (Genin and Denny, 2012; Peeters et al., 2013; Huet, 2014; Yuliar et al., 2015). A putative plant resistance gene to this pathogen is *WAT1* (for *Walls Are Thin1*), required for secondary cell-wall deposition in *M. truncatula* (Ranocha et al., 2013). WAT1 is involved in auxin homeostasis in relation to vacuolar auxin transport, and the inactivation of *WAT1* confers a broad spectrum resistance to several vascular pathogenic bacteria including *R. solanacearum* and *Xanthomonas campestris* (Denancé et al., 2013). Transcriptomic and metabolomic analyses demonstrated a repression of several genes linked to auxin metabolism in *wat1* mutant roots, correlated with a decrease of a major form of auxin (indole glucosinolate) and to a reduction in the amount of the auxin precursor tryptophan. Interestingly, crossing of the *wat1* mutant with a *trp5* mutant carrying a mutation of an anthranilate synthase (ASA1) provokes an over-accumulation of tryptophan, and restores *wat1* susceptibility to *R. solanacearum*. Altogether, these results suggest a positive role of auxins in secondary wall formation, as well as in the susceptibility to pathogenic *R. solanacearum* infections. In addition to auxins, an upregulation of cytokinin response genes, such as *CKX* and a few *RRAs*, was identified in *M. truncatula* by a transcriptomic approach in response to *R. solanacearum* (Moreau et al., 2014). This notably includes the *MtRRA4* Response Regulator, which is transcriptionally upregulated by both cytokinins and *Ralstonia*, depending on the MtCRE1 cytokinin receptor. Accordingly, the *cre1* mutant shows an increased resistance to *R. solanacearum*, indicating a role of cytokinins in promoting root susceptibility to the pathogen.

## Concluding Remarks

Rhizospheric beneficial and detrimental microbes penetrate into root systems and tissues and trigger major modifications at organ, cellular and molecular levels, notably through modifications of developmental phytohormonal balances. The **Table 1** summarizes roles of auxins and cytokinins in different root–microbe interactions. A main feature is that as auxins and cytokinins are critical to regulate cell division and differentiation, these hormones are therefore tightly associated with the formation of new organs such as lateral roots, nodules on legume roots in response to rhizobia, as well as galls for example in response to *A. tumefaciens* or to *P. brassicae* infection (**Figure 1**; **Table 1**). Most of the rhizospheric microbes, either symbiotic or pathogenic, affect the root system architecture, generally by altering lateral root formation and/or root hair growth (e.g., *Laccaria bicolor* and rhizobia; **Table 1**). In agreement, NFs and Myc Factors, and the associated N_2_-fixing and AM symbionts, induce lateral root development as part of the symbiotic response (Oláh et al., 2005; Maillet et al., 2011) (**Figure 1**). However, links likely existing with hormones controlling lateral root development, and notably auxins, remain to be identified. Auxins, cytokinins, and their associated signaling pathways are also required for inducing root cortical cell divisions, either in legume plants to generate nodule primordia in response to rhizobia, or in *Brassicaceae* plants to form galls in response to the *P. brassicae* pathogen (**Figure 1**; **Table 1**). Interestingly, in these distantly related host plants, cortical cell divisions are similarly associated with the accumulation of naringenin and kaempferol flavonoids. Results reported in this review highlight a positive role of auxins and cytokinins in plant root susceptibility to pathogens, except for *F. oxysporum*, as well as to rhizobia symbiotic bacteria (**Table 1**). Using competitors of root cortical colonization such as endophytic fungi and/or rhizobia may then be a strategy to prevent root pathogen colonization. Understanding the different pathways used by beneficial and detrimental microbes to alter root system development, invade the root cortex, and sometimes to generate new organs, is a crucial challenge to develop integrated strategies to promote crop protection without altering symbiotic capacities, in the frame of sustainable agriculture and agro-ecology practices.

**Table 1:** Summary of known functions of auxins and cytokinins in various root–microbe interactions.

	Root symbionts				
	Fungal symbionts		Bacterial symbionts		Root pathogens
	ECM fungi	AM fungi	Rhizobia	*Frankia*	
Auxins	– Auxin production by fungi stimulates lateral root and root hair formation – Ethylene production by fungi activates plant auxin production – Auxin response (DR5, GH3) and transport (PIN) associated to infections	– Auxin response (GH3, DR5) associated to infections – miR393 downregulates auxin perception and negatively regulates arbuscule formation	– Auxin production by rhizobia; ectopic auxin overproduction promotes nodulation – Auxin response (GH3, Aux/IAA, SAUR, DR5) associated to epidermal infections and cortical cell divisions/nodule primordia – A local inhibition of PAT (and *PIN* gene expression) is associated to rhizobial infections – The symbiotic inhibition of PAT depends on flavonoids and on cytokinins (see below) – ARF16a positively regulates infections in the root epidermis	– Auxin response (AUX/IAA) is associated to *Frankia* infections – Auxin influx (AUX1) is associated to infected cells – Auxin induces the degradation of Aux/IAA (e.g., IAA7) auxin-signaling repressors and inhibits nodulation	– Many root pathogens produce auxin and agrobacteria transfer DNA encoding auxin-synthesis genes – Auxin response (GH3) is associated to *P. brassicae* gall formation (in relation to flavonoid accumulation?) – Auxin application may lead to the resistance to *F. oxysporum* – WAT1 auxin-deficient plants are more resistant to *R. solanacearum*
Cytokinins	– Cytokinin production by fungi (effect on roots?)	– ? (some changes in cytokinin contents are observed)	– Cytokinin production by rhizobia; ectopic cytokinin overproduction promotes nodulation but minor effects of cytokinin deficient strains on nodulation – Cytokinin response (Type A RRs, TCS) associated to nodule initiation, with cortical cell divisions and/or epidermal infections – Cytokinins rapidly regulate several Nod factor signaling genes – Cytokinin perception mutants (CRE1/LHK1) display a reduced nodulation and a LHK1gof variant promotes spontaneous nodulation (i.e., nodule organogenesis); redundant roles of other cytokinin receptors – CRE1-cytokinin signaling acts upstream of specific flavonoid accumulation and of auxin transport inhibition and response – Cytokinins negatively regulate infections and/or Nod factor signaling – Cytokinins also likely regulate nodule differentiation and nitrogen fixation	– ?	– Many root pathogens produce cytokinins and agrobacteria transfer DNA encoding cytokinin-synthesis genes – Cytokinin response (Type A RR) and metabolism are associated to *P. brassicae* gall formation (in relation to flavonoid accumulation?) – Mutation of the CRE1 cytokinin receptor promotes resistance to *A. euteiches* and *R. solanacearum*

*The columns indicate the different symbiotic and pathogenic interactions and the rows indicate the phytohormones auxins and cytokinins. ECM, EctoMycorrhiza; AM, Arbuscular Mycorrhiza; GH3, Gretchen Hagen3; DR5, Auxin-response reporter; ARF, Auxin Response Factor; Aux/IAA, Indole-3-Acetic Acid Auxin-response protein; SAUR, Small Auxin Up RNA; PIN, Pin-formed protein; PAT, Polar Auxin Transport; RR, Response Regulator cytokinin response gene; CRE1, Cytokinin Response 1 receptor; LHK1, Lotus Histidine Kinase cytokinin receptor; TCS, Two Component output Sensor cytokinin-response reporter; WAT1, Walls Are Thin1; gof, gain of function.*

## Author Contributions

SB and FF wrote the manuscript with inputs from CFF, and CFF produced the figures.

## Conflict of Interest Statement

The authors declare that the research was conducted in the absence of any commercial or financial relationships that could be construed as a potential conflict of interest.

## References

[B1] Akiyoshi D. E. Klee H. Amasino R. M. Nester E. W. Gordon M. P. (1984). T-DNA of *Agrobacterium tumefaciens* encodes an enzyme of cytokinin biosynthesis. *Proc. Natl. Acad. Sci. U.S.A.* 81 5994–5998. 10.1073/pnas.81.19.5994 6091129 PMC391845

[B2] Akiyoshi D. E. Regier D. A. Gordon M. P. (1987). Cytokinin production by *Agrobacterium* and *Pseudomonas* spp. *J. Bacteriol.* 169 4242–4248. 3624204 10.1128/jb.169.9.4242-4248.1987 PMC213736

[B3] Allen E. K. Allen O. N. Newman A. S. (1953). Pseudonodulation of leguminous plants induced by 2-bromo-3,5-dichlorobenzoic acid. *Am. J. Bot.* 40 429–435.

[B4] Allen M. F. Moore T. S. Jr. Christensen M. (1980). Phytohormone changes in *Bouteloua gracilis* infected by vesicular–arbuscular mycorrhizae: I. Cytokinin increases in the host plant. *Can. J. Bot.* 58 371–374. 10.1139/b80-038

[B5] Anderson I. C. Cairney J. W. G. (2007). Ectomycorrhizal fungi: exploring the mycelial frontier. *FEMS Microbiol. Rev.* 31 388–406. 10.1111/j.1574-6976.2007.00073.x 17466031

[B6] Ando S. Tsushima S. Tagiri A. Kamachi S. Konagaya K.-I. Hagio T. (2006). Increase in BrAO1 gene expression and aldehyde oxidase activity during clubroot development in Chinese cabbage (*Brassica rapa* L.). *Mol. Plant Pathol.* 7 223–234. 10.1111/j.1364-3703.2006.00333.x 20507442

[B7] Argueso C. T. Ferreira F. J. Kieber J. J. (2009). Environmental perception avenues: the interaction of cytokinin and environmental response pathways. *Plant Cell Environ.* 32 1147–1160. 10.1111/j.1365-3040.2009.01940.x 19183294

[B8] Ariel F. Brault-Hernandez M. Laffont C. Huault E. Brault M. Plet J. (2012). Two direct targets of cytokinin signaling regulate symbiotic nodulation in *Medicago truncatula*. *Plant Cell* 24 3838–3852. 10.1105/tpc.112.103267 23023168 PMC3480306

[B9] Barker S. J. Tagu D. (2000). The roles of auxins and cytokinins in mycorrhizal symbioses. *J. Plant Growth Regul.* 19 144–154. 11038224 10.1007/s003440000021

[B10] Bauer P. Ratet P. Crespi M. D. Schultze M. Kondorosi A. (1996). Nod factors and cytokinins induce similar cortical cell division, amyloplast deposition and MsEnod12A expression patterns in alfalfa roots. *Plant J.* 10 91–105. 10.1046/j.1365-313X.1996.10010091.x

[B11] Bianco C. Defez R. (2010). Improvement of phosphate solubilization and *Medicago* plant yield by an indole-3-acetic acid-overproducing strain of *Sinorhizobium meliloti*. *Appl. Environ. Microbiol.* 76 4626–4632. 10.1128/AEM.02756-09 20511434 PMC2901742

[B12] Boivin S. Kazmierczak T. Brault M. Wen J. Gamas P. Mysore K. S. (2016). Different cytokinin CHK receptors regulate nodule initiation as well as later nodule developmental stages in *Medicago truncatula*. *Plant Cell Environ.* 10.1111/pce.12779 [Epub ahead of print]. 27341695

[B13] Bonfante P. Requena N. (2011). Dating in the dark: how roots respond to fungal signals to establish arbuscular mycorrhizal symbiosis. *Curr. Opin. Plant Biol.* 14 451–457. 10.1016/j.pbi.2011.03.014 21489861

[B14] Bonhomme M. André O. Badis Y. Ronfort J. Burgarella C. Chantret N. (2014). High-density genome-wide association mapping implicates an F-box encoding gene in *Medicago truncatula* resistance to *Aphanomyces euteiches*. *New Phytol.* 201 1328–1342. 10.1111/nph.12611 24283472

[B15] Boot K. J. M. van Brussel A. A. N. Tak T. Spaink H. P. Kijne J. W. (1999). Lipochitin oligosaccharides from *Rhizobium leguminosarum* bv. viciae reduce auxin transport capacity in *Vicia sativa* subsp. nigra roots. *Mol. Plant Microbe Interact.* 12 839–844. 10.1094/MPMI.1999.12.10.839

[B16] Breakspear A. Liu C. Roy S. Stacey N. Rogers C. Trick M. (2014). The root hair “infectome” of *Medicago truncatula* uncovers changes in cell cycle genes and reveals a requirement for Auxin signaling in rhizobial infection. *Plant Cell* 26 4680–4701. 10.1105/tpc.114.133496 25527707 PMC4311213

[B17] Bustos-Sanmamed P. Tovar-Méndez A. Crespi M. Sato S. Tabata S. Becana M. (2011). Regulation of nonsymbiotic and truncated hemoglobin genes of *Lotus japonicus* in plant organs and in response to nitric oxide and hormones. *New Phytol.* 189 765–776. 10.1111/j.1469-8137.2010.03527.x 21073469

[B18] Camerini S. Senatore B. Lonardo E. Imperlini E. Bianco C. Moschetti G. (2008). Introduction of a novel pathway for IAA biosynthesis to rhizobia alters vetch root nodule development. *Arch. Microbiol.* 190 67–77. 10.1007/s00203-008-0365-7 18415080

[B19] Champion A. Lucas M. Tromas A. Vaissayre V. Crabos A. Diédhiou I. (2015). Inhibition of auxin signaling in Frankia species-infected cells in *Casuarina glauca* nodules leads to increased nodulation. *Plant Physiol.* 167 1149–1157. 10.1104/pp.114.255307 25627215 PMC4348781

[B20] Chang L. Ramireddy E. Schmülling T. (2013). Lateral root formation and growth of *Arabidopsis* is redundantly regulated by cytokinin metabolism and signalling genes. *J. Exp. Bot.* 64 5021–5032. 10.1093/jxb/ert291 24023250 PMC3830484

[B21] Chen D. S. Liu C. W. Roy S. Cousins D. Stacey N. Murray J. D. (2015). Identification of a core set of rhizobial infection genes using data from single cell-types. *Front. Plant Sci.* 6:575 10.3389/fpls.2015.00575 PMC4517396 26284091

[B22] Chen Y. Chen W. Li X. Jiang H. Wu P. Xia K. (2014). Knockdown of LjIPT3 influences nodule development in *Lotus japonicus*. *Plant Cell Physiol.* 55 183–193. 10.1093/pcp/pct171 24285753

[B23] Chen Y. C. Kidd B. N. Carvalhais L. C. Schenk P. M. (2014). Molecular defense responses in roots and the rhizosphere against *Fusarium oxysporum*. *Plant Signal. Behav.* 9:e977710 10.4161/15592324.2014.977710 PMC4623376 25482759

[B24] Cooper J. B. Long S. R. (1994). Morphogenetic rescue of *Rhizobium meliloti* nodulation mutants by trans-zeatin secretion. *Plant Cell* 6 215–225. 10.1105/tpc.6.2.215 12244237 PMC160428

[B25] Costantino P. Hooykaas P. J. den Dulk-Ras H. Schilperoort R. A. (1980). Tumor formation and rhizogenicity of *Agrobacterium* rhizogenes carrying Ti plasmids. *Gene* 11 79–87. 10.1016/0378-1119(80)90088-8 7439687

[B26] De Vleesschauwer D. Xu J. Höfte M. (2014). Making sense of hormone-mediated defense networking: from rice to *Arabidopsis*. *Front. Plant Sci.* 5:611 10.3389/fpls.2014.00611 PMC4227482 25426127

[B27] de Zelicourt A. Al-Yousif M. Hirt H. (2013). Rhizosphere microbes as essential partners for plant stress tolerance. *Mol. Plant* 6 242–245. 10.1093/mp/sst028 23475999

[B28] Defez R. Esposito R. Angelini C. Bianco C. (2016). Overproduction of indole-3-acetic acid in free-living rhizobia induces transcriptional changes resembling those occurring in nodule bacteroids. *Mol. Plant Microbe Interact.* 29 484–495. 10.1094/MPMI-01-16-0010-R 27003799

[B29] Dehio C. de Bruijn F. J. (1992). The early nodulin gene SrEnod2 from *Sesbania rostrata* is inducible by cytokinin. *Plant J.* 2 117–128. 10.1046/j.1365-313X.1992.t01-51-00999.x 1303791

[B30] Denancé N. Ranocha P. Oria N. Barlet X. Rivière M.-P. Yadeta K. A. (2013). *Arabidopsis* wat1 (walls are thin1)-mediated resistance to the bacterial vascular pathogen, *Ralstonia solanacearum*, is accompanied by cross-regulation of salicylic acid and tryptophan metabolism. *Plant J.* 73 225–239. 10.1111/tpj.12027 22978675

[B31] Dénarié J. Debellé F. Rosenberg C. (1992). Signaling and host range variation in nodulation. *Annu. Rev. Microbiol.* 46 497–531. 10.1146/annurev.mi.46.100192.002433 1444265

[B32] Desbrosses G. J. Stougaard J. (2011). Root nodulation: a paradigm for how plant-microbe symbiosis influences host developmental pathways. *Cell Host Microbe* 10 348–358. 10.1016/j.chom.2011.09.005 22018235

[B33] Devos S. Laukens K. Deckers P. Van Der Straeten D. Beeckman T. Inzé D. (2006). A hormone and proteome approach to picturing the initial metabolic events during *Plasmodiophora brassicae* infection on *Arabidopsis*. *Mol. Plant Microbe Interact.* 19 1431–1443. 10.1094/MPMI-19-1431 17153927

[B34] Diagne N. Diouf D. Svistoonoff S. Kane A. Noba K. Franche C. (2013). Casuarina in Africa: distribution, role and importance of arbuscular mycorrhizal, ectomycorrhizal fungi and Frankia on plant development. *J. Environ. Manage.* 128 204–209. 10.1016/j.jenvman.2013.05.009 23747371

[B35] Djébali N. Jauneau A. Ameline-Torregrosa C. Chardon F. Jaulneau V. Mathé C. (2009). Partial resistance of *Medicago truncatula* to *Aphanomyces euteiches* is associated with protection of the root stele and is controlled by a major QTL rich in proteasome-related genes. *Mol. Plant Microbe Interact.* 22 1043–1055. 10.1094/MPMI-22-9-1043 19656040

[B36] Dowd C. Wilson I. W. McFadden H. (2004). Gene expression profile changes in cotton root and hypocotyl tissues in response to infection with *Fusarium oxysporum* f. sp. vasinfectum. *Mol. Plant Microbe Interact.* 17 654–667. 10.1094/MPMI.2004.17.6.654 15195948

[B37] Estruch J. J. Schell J. Spena A. (1991). The protein encoded by the rolB plant oncogene hydrolyses indole glucosides. *EMBO J.* 10 3125–3128. 1915286 10.1002/j.1460-2075.1991.tb04873.x PMC453033

[B38] Etemadi M. Gutjahr C. Couzigou J. M. Zouine M. Lauressergues D. Timmers A. (2014). Auxin perception is required for arbuscule development in arbuscular mycorrhizal symbiosis. *Plant Physiol.* 166 281–292. 10.1104/pp.114.246595 25096975 PMC4149713

[B39] Felten J. Kohler A. Morin E. Bhalerao R. P. Palme K. Martin F. (2009). The ectomycorrhizal fungus *Laccaria bicolor* stimulates lateral root formation in poplar and *Arabidopsis* through auxin transport and signaling. *Plant Physiol.* 151 1991–2005. 10.1104/pp.109.147231 19854859 PMC2785963

[B40] Ferguson B. J. Indrasumunar A. Hayashi S. Lin M.-H. Lin Y.-H. Reid D. E. (2010). Molecular analysis of legume nodule development and autoregulation. *J. Integr. Plant Biol.* 52 61–76. 10.1111/j.1744-7909.2010.00899.x 20074141

[B41] Foo E. (2013). Auxin influences strigolactones in pea mycorrhizal symbiosis. *J. Plant Physiol.* 170 523–528. 10.1016/j.jplph.2012.11.002 23219475

[B42] Foo E. Ross J. J. Jones W. T. Reid J. B. (2013). Plant hormones in arbuscular mycorrhizal symbioses: an emerging role for gibberellins. *Ann. Bot.* 111 769–779. 10.1093/aob/mct041 23508650 PMC3631329

[B43] Franssen H. J. Vijn I. Yang W. C. Bisseling T. (1992). Developmental aspects of the *Rhizobium*-legume symbiosis. *Plant Mol. Biol.* 19 89–107. 10.1007/BF00015608 1600171

[B44] Fusconi A. (2014). Regulation of root morphogenesis in arbuscular mycorrhizae: what role do fungal exudates, phosphate, sugars and hormones play in lateral root formation? *Ann. Bot.* 113 19–33. 10.1093/aob/mct258 24227446 PMC3864729

[B45] Gaulin E. Jacquet C. Bottin A. Dumas B. (2007). Root rot disease of legumes caused by *Aphanomyces euteiches*. *Mol. Plant Pathol.* 8 539–548. 10.1111/j.1364-3703.2007.00413.x 20507520

[B46] Genin S. Denny T. P. (2012). Pathogenomics of the *Ralstonia solanacearum* species complex. *Annu. Rev. Phytopathol.* 50 67–89. 10.1146/annurev-phyto-081211-173000 22559068

[B47] Genre A. Chabaud M. Balzergue C. Puech-Pagès V. Novero M. Rey T. (2013). Short-chain chitin oligomers from arbuscular mycorrhizal fungi trigger nuclear Ca2+ spiking in *Medicago truncatula* roots and their production is enhanced by strigolactone. *New Phytol.* 198 190–202. 10.1111/nph.12146 23384011

[B48] Geurts R. Lillo A. Bisseling T. (2012). Exploiting an ancient signalling machinery to enjoy a nitrogen fixing symbiosis. *Curr. Opin. Plant Biol.* 15 438–443. 10.1016/j.pbi.2012.04.004 22633856

[B49] Gonzalez-Rizzo S. Crespi M. Frugier F. (2006). The *Medicago truncatula* CRE1 cytokinin receptor regulates lateral root development and early symbiotic interaction with *Sinorhizobium meliloti*. *Plant Cell* 18 2680–2693. 10.1105/tpc.106.043778 17028204 PMC1626621

[B50] Gough C. Cullimore J. (2011). Lipo-chitooligosaccharide signaling in endosymbiotic plant-microbe interactions. *Mol. Plant Microbe Interact.* 24 867–878. 10.1094/MPMI-01-11-0019 21469937

[B51] Gourion B. Berrabah F. Ratet P. Stacey G. (2015). *Rhizobium*-legume symbioses: the crucial role of plant immunity. *Trends Plant Sci.* 20 186–194. 10.1016/j.tplants.2014.11.008 25543258

[B52] Grover A. Mittal D. Negi M. Lavania D. (2013). Generating high temperature tolerant transgenic plants: achievements and challenges. *Plant Sci.* 20 38–47. 10.1016/j.plantsci.2013.01.005 23498861

[B53] Gutjahr C. Parniske M. (2013). Cell and developmental biology of arbuscular mycorrhiza symbiosis. *Annu. Rev. Cell Dev. Biol.* 29 593–617. 10.1146/annurev-cellbio-101512-122413 24099088

[B54] Hanlon M. T. Coenen C. (2011). Genetic evidence for auxin involvement in arbuscular mycorrhiza initiation. *New Phytol.* 189 701–709. 10.1111/j.1469-8137.2010.03567.x 21091696

[B55] Held M. Hou H. Miri M. Huynh C. Ross L. Hossain M. S. (2014). *Lotus japonicus* cytokinin receptors work partially redundantly to mediate nodule formation. *Plant Cell* 26 678–694. 10.1105/tpc.113.119362 24585837 PMC3967033

[B56] Heyl A. Brault M. Frugier F. Kuderova A. Lindner A.-C. Motyka V. (2013). Nomenclature for members of the two-component signaling pathway of plants. *Plant Physiol.* 161 1063–1065. 10.1104/pp.112.213207 23324541 PMC3585578

[B57] Huet G. (2014). Breeding for resistances to *Ralstonia solanacearum*. *Front. Plant Sci.* 5:715 10.3389/fpls.2014.00715 PMC4264415 25566289

[B58] Hwang H.-H. Wang M.-H. Lee Y.-L. Tsai Y.-L. Li Y.-H. Yang F.-J. (2010). *Agrobacterium*-produced and exogenous cytokinin-modulated *Agrobacterium*-mediated plant transformation. *Mol. Plant Pathol.* 11 677–690. 10.1111/j.1364-3703.2010.00637.x 20696005 PMC6640272

[B59] Hwang S.-F. Strelkov S. E. Feng J. Gossen B. D. Howard R. J. (2012). *Plasmodiophora brassicae*: a review of an emerging pathogen of the Canadian canola (*Brassica napus*) crop. *Mol. Plant Pathol.* 13 105–113. 10.1111/j.1364-3703.2011.00729.x 21726396 PMC6638701

[B60] Jardineau M. F. Boivin S. Rodde N. Catrice O. Kisiala A. Lepage A. (2016). A laser dissection-RNAseq analysis highlights the activation of cytokinin pathways by Nod factors in the *Medicago truncatula* root epidermis. *Plant Physiol.* 171 2256–2276. 10.1104/pp.16.00711 27217496 PMC4936592

[B61] Jentschel K. Thiel D. Rehn F. Ludwig-Müller J. (2007). Arbuscular mycorrhiza enhances auxin levels and alters auxin biosynthesis in *Tropaeolum majus* during early stages of colonization. *Physiol. Plant.* 129 320–333. 10.1111/j.1399-3054.2006.00812.x

[B62] Jiménez-Zurdo J. I. Frugier F. Crespi M. D. Kondorosi A. (2000). Expression profiles of 22 novel molecular markers for organogenetic pathways acting in alfalfa nodule development. *Mol. Plant Microbe Interact.* 13 96–106. 10.1094/MPMI.2000.13.1.96 10656590

[B63] Kaló P. Gleason C. Edwards A. Marsh J. Mitra R. M. Hirsch S. (2005). Nodulation signaling in legumes requires NSP2, a member of the GRAS family of transcriptional regulators. *Science* 308 1786–1789. 10.1126/science.1110951 15961668

[B64] Karabaghli C. Frey-Klett P. Sotta B. Bonnet M. Le Tacon F. (1998). In vitro effects of *Laccaria bicolor* S238 N and *Pseudomonas* fluorescens strain BBc6 on rooting of de-rooted shoot hypocotyls of *Norway spruce*. *Tree Physiol.* 18 103–111. 10.1093/treephys/18.2.103 12651394

[B65] Kisiala A. Laffont C. Emery R. J. N. Frugier F. (2013). Bioactive cytokinins are selectively secreted by *Sinorhizobium meliloti* nodulating and nonnodulating strains. *Mol. Plant Microbe Interact.* 26 1225–1231. 10.1094/MPMI-02-13-0054-R 24001254

[B66] Kutáček M. Rovenská J. (1991). Auxin synthesis in *Agrobacterium tumefaciens* and *A. tumefaciens*-transformed plant tissue. *Plant Growth Regul.* 10 313–327. 10.1007/BF00024591

[B67] Laffont C. Rey T. André O. Novero M. Kazmierczak T. Debellé F. (2015). The CRE1 cytokinin pathway is differentially recruited depending on *Medicago truncatula* root environments and negatively regulates resistance to a pathogen. *PLoS ONE* 10:e0116819 10.1371/journal.pone.0116819 PMC4285552 25562779

[B68] Lahlali R. McGregor L. Song T. Gossen B. D. Narisawa K. Peng G. (2014). *Heteroconium chaetospira* induces resistance to clubroot via upregulation of host genes involved in jasmonic acid, ethylene, and auxin biosynthesis. *PLoS ONE* 9:e94144 10.1371/journal.pone.0094144 PMC3979836 24714177

[B69] Laplaze L. Lucas M. Champion A. (2015). Rhizobial root hair infection requires auxin signaling. *Trends Plant Sci.* 20 332–334. 10.1016/j.tplants.2015.04.004 25920666

[B70] Lee C.-W. Efetova M. Engelmann J. C. Kramell R. Wasternack C. Ludwig-Müller J. (2009). *Agrobacterium tumefaciens* promotes tumor induction by modulating pathogen defense in *Arabidopsis thaliana*. *Plant Cell* 21 2948–2962. 10.1105/tpc.108.064576 19794116 PMC2768927

[B71] Liao D. Chen X. Chen A. Wang H. Liu J. Liu J. (2015). The characterization of six auxin-induced tomato GH3 genes uncovers a member, SlGH3.4, strongly responsive to arbuscular mycorrhizal symbiosis. *Plant Cell Physiol.* 56 674–687. 10.1093/pcp/pcu212 25535196

[B72] Libbenga K. R. van Iren F. Bogers R. J. Schraag-Lamers M. F. (1973). The role of hormones and gradients in the initiation of cortex proliferation and nodule formation in *Pisum sativum* L. *Planta* 114 29–39. 10.1007/BF00390282 24458662

[B73] Liu C.-W. Breakspear A. Roy S. Murray J. D. (2015). Cytokinin responses counterpoint auxin signaling during rhizobial infection. *Plant Signal. Behav.* 10:e1019982 10.1080/15592324.2015.1019982 PMC4623047 26176899

[B74] Liu S. T. Kado C. I. (1979). Indoleacetic acid production: a plasmid function of *Agrobacterium tumefaciens* C58. *Biochem. Biophys. Res. Commun.* 90 171–178. 10.1016/0006-291X(79)91605-X 496970

[B75] Lohar D. P. Schaff J. E. Laskey J. G. Kieber J. J. Bilyeu K. D. Bird D. M. (2004). Cytokinins play opposite roles in lateral root formation, and nematode and rhizobial symbioses. *Plant J.* 38 203–214. 10.1111/j.1365-313X.2004.02038.x 15078325

[B76] Lyons R. Stiller J. Powell J. Rusu A. Manners J. M. Kazan K. (2015). *Fusarium oxysporum* triggers tissue-specific transcriptional reprogramming in *Arabidopsis thaliana*. *PLoS ONE* 10:e0121902 10.1371/journal.pone.0121902 PMC4388846 25849296

[B77] Maillet F. Poinsot V. André O. Puech-Pagès V. Haouy A. Gueunier M. (2011). Fungal lipochitooligosaccharide symbiotic signals in arbuscular mycorrhiza. *Nature* 469 58–63. 10.1038/nature09622 21209659

[B78] Mathesius U. Charon C. Rolfe B. G. Kondorosi A. Crespi M. (2000). Temporal and spatial order of events during the induction of cortical cell divisions in white clover by *Rhizobium leguminosarum* bv. trifolii inoculation or localized cytokinin addition. *Mol. Plant Microbe Interact.* 13 617–628. 10.1094/MPMI.2000.13.6.617 10830261

[B79] Mathesius U. Schlaman H. R. Spaink H. P. Of Sautter C. Rolfe B. G. Djordjevic M. A. (1998). Auxin transport inhibition precedes root nodule formation in white clover roots and is regulated by flavonoids and derivatives of chitin oligosaccharides. *Plant J.* 14 23–34. 10.1046/j.1365-313X.1998.00090.x 15494052

[B80] Moreau S. Fromentin J. Vailleau F. Vernié T. Huguet S. Balzergue S. (2014). The symbiotic transcription factor MtEFD and cytokinins are positively acting in the *Medicago truncatula* and *Ralstonia solanacearum* pathogenic interaction. *New Phytol.* 201 1343–1357. 10.1111/nph.12636 24325235

[B81] Morrison E. N. Knowles S. Hayward A. Thorn R. G. Saville B. J. Emery R. J. N. (2015). Detection of phytohormones in temperate forest fungi predicts consistent abscisic acid production and a common pathway for cytokinin biosynthesis. *Mycologia* 107 245–257. 10.3852/14-157 25572099

[B82] Mortier V. Wasson A. Jaworek P. De Keyser A. Decroos M. Holsters M. (2014). Role of LONELY GUY genes in indeterminate nodulation on *Medicago truncatula*. *New Phytol.* 202 582–593. 10.1111/nph.12681 24443934

[B83] Murray J. D. Karas B. J. Sato S. Tabata S. Amyot L. Szczyglowski K. (2007). A cytokinin perception mutant colonized by *Rhizobium* in the absence of nodule organogenesis. *Science* 315 101–104. 10.1126/science.1132514 17110535

[B84] Naseem M. Dandekar T. (2012). The role of auxin-cytokinin antagonism in plant-pathogen interactions. *PLoS Pathog.* 8:e1003026 10.1371/journal.ppat.1003026 PMC3510258 23209407

[B85] Ng J. L. P. Hassan S. Truong T. T. Hocart C. H. Laffont C. Frugier F. (2015). Flavonoids and auxin transport inhibitors rescue symbiotic nodulation in the *Medicago truncatula* cytokinin perception mutant cre1. *Plant Cell* 27 2210–2226. 10.1105/tpc.15.00231 26253705 PMC4568502

[B86] Oláh B. Brière C. Bécard G. Dénarié J. Gough C. (2005). Nod factors and a diffusible factor from arbuscular mycorrhizal fungi stimulate lateral root formation in *Medicago truncatula* via the DMI1/DMI2 signalling pathway. *Plant J.* 44 195–207. 10.1111/j.1365-313X.2005.02522.x 16212600

[B87] Oldroyd G. E. D. (2013). Speak, friend, and enter: signalling systems that promote beneficial symbiotic associations in plants. *Nat. Rev. Microbiol.* 11 252–263. 10.1038/nrmicro2990 23493145

[B88] Oldroyd G. E. D. Murray J. D. Poole P. S. Downie J. A. (2011). The rules of engagement in the legume-rhizobial symbiosis. *Annu. Rev. Genet.* 45 119–144. 10.1146/annurev-genet-110410-132549 21838550

[B89] Op den Camp R. H. M. De Mita S. Lillo A. Cao Q. Limpens E. Bisseling T. (2011). A phylogenetic strategy based on a legume-specific whole genome duplication yields symbiotic cytokinin type-A response regulators1. *Plant Physiol.* 157 2013–2022. 10.1104/pp.111.187526 22034625 PMC3327194

[B90] Pacios-Bras C. Schlaman H. R. M. Boot K. Admiraal P. Langerak J. M. Stougaard J. (2003). Auxin distribution in *Lotus japonicus* during root nodule development. *Plant Mol. Biol.* 52 1169–1180. 10.1023/B:PLAN.0000004308.78057.f5 14682616

[B91] Päsold S. Siegel I. Seidel C. Ludwig-Müller J. (2010). Flavonoid accumulation in *Arabidopsis thaliana* root galls caused by the obligate biotrophic pathogen *Plasmodiophora brassicae*. *Mol. Plant Pathol.* 11 545–562. 10.1111/j.1364-3703.2010.00628.x 20618711 PMC6640481

[B92] Peeters N. Guidot A. Vailleau F. Valls M. (2013). *Ralstonia solanacearum*, a widespread bacterial plant pathogen in the post-genomic era. *Mol. Plant Pathol.* 14 651–662. 10.1111/mpp.12038 23718203 PMC6638647

[B93] Péret B. Swarup R. Jansen L. Devos G. Auguy F. Collin M. (2007). Auxin influx activity is associated with Frankia infection during actinorhizal nodule formation in *Casuarina glauca*. *Plant Physiol.* 144 1852–1862. 10.1104/pp.107.101337 17556507 PMC1949887

[B94] Perret X. Staehelin C. Broughton W. J. (2000). Molecular basis of symbiotic promiscuity. *Microbiol. Mol. Biol. Rev.* 64 180–201. 10.1128/MMBR.64.1.180-201.2000 10704479 PMC98991

[B95] Perrine-Walker F. Doumas P. Lucas M. Vaissayre V. Beauchemin N. J. Band L. R. (2010). Auxin carriers localization drives auxin accumulation in plant cells infected by Frankia in *Casuarina glauca* actinorhizal nodules. *Plant Physiol.* 154 1372–1380. 10.1104/pp.110.163394 20826704 PMC2971613

[B96] Peterson R. L. Massicotte H. B. (2004). Exploring structural definitions of mycorrhizas, with emphasis on nutrient-exchange interfaces. *Can. J. Bot.* 82 1074–1088. 10.1139/b04-071

[B97] Phillips D. A. Torrey J. G. (1972). Studies on cytokinin production by *Rhizobium*. *Plant Physiol.* 49 11–15. 10.1104/pp.49.1.11 16657888 PMC365892

[B98] Pieterse C. M. J. Van der Does D. Zamioudis C. Leon-Reyes A. Van Wees S. C. M. (2012). Hormonal modulation of plant immunity. *Annu. Rev. Cell Dev. Biol.* 28 489–521. 10.1146/annurev-cellbio-092910-154055 22559264

[B99] Plet J. Wasson A. Ariel F. Le Signor C. Baker D. Mathesius U. (2011). MtCRE1-dependent cytokinin signaling integrates bacterial and plant cues to coordinate symbiotic nodule organogenesis in *Medicago truncatula*. *Plant J.* 65 622–633. 10.1111/j.1365-313X.2010.04447.x 21244535

[B100] Podlešáková K. Fardoux J. Patrel D. Bonaldi K. Novák O. Strnad M. (2013). Rhizobial synthesized cytokinins contribute to but are not essential for the symbiotic interaction between photosynthetic Bradyrhizobia and *Aeschynomene legumes*. *Mol. Plant Microbe Interact.* 26 1232–1238. 10.1094/MPMI-03-13-0076-R 23777431

[B101] Ranocha P. Dima O. Nagy R. Felten J. Corratgé-Faillie C. Novák O. (2013). *Arabidopsis* WAT1 is a vacuolar auxin transport facilitator required for auxin homoeostasis. *Nat. Commun.* 4 2625 10.1038/ncomms3625 PMC3826630 24129639

[B102] Raudaskoski M. Kothe E. (2015). Novel findings on the role of signal exchange in arbuscular and ectomycorrhizal symbioses. *Mycorrhiza* 25 243–252. 10.1007/s00572-014-0607-2 25260351

[B103] Reid D. E. Heckmann A. B. Novak O. Kelly S. Stougaard J. (2016). CYTOKININ OXIDASE/DESHYDROGENASE3 maintains cytokinin homeostasis during root and nodule development in *Lotus japonicus*. *Plant Physiol.* 170 1060–1074. 10.1104/pp.15.00650 26644503 PMC4734552

[B104] Rightmyer A. P. Long S. R. (2011). Pseudonodule formation by wild-type and symbiotic mutant *Medicago truncatula* in response to auxin transport inhibitors. *Mol. Plant Microbe Interact.* 24 1372–1384. 10.1094/MPMI-04-11-0103 21809981

[B105] Robert-Seilaniantz A. Grant M. Jones J. D. G. (2011). Hormone crosstalk in plant disease and defense: more than just jasmonate-salicylate antagonism. *Annu. Rev. Phytopathol.* 49 317–343. 10.1146/annurev-phyto-073009-114447 21663438

[B106] Ross E. J. H. Stone J. M. Elowsky C. G. Arredondo-Peter R. Klucas R. V. Sarath G. (2004). Activation of the *Oryza sativa* non-symbiotic haemoglobin-2 promoter by the cytokinin-regulated transcription factor, ARR1. *J. Exp. Bot.* 55 1721–1731. 10.1093/jxb/erh211 15258171

[B107] Roux B. Rodde N. Jardinaud M.-F. Timmers T. Sauviac L. Cottret L. (2014). An integrated analysis of plant and bacterial gene expression in symbiotic root nodules using laser-capture microdissection coupled to RNA sequencing. *Plant J.* 77 817–837. 10.1111/tpj.12442 24483147

[B108] Santi C. Bogusz D. Franche C. (2013). Biological nitrogen fixation in non-legume plants. *Ann. Bot.* 111 743–767. 10.1093/aob/mct048 23478942 PMC3631332

[B109] Sasaki T. Suzaki T. Soyano T. Kojima M. Sakakibara H. Kawaguchi M. (2014). Shoot-derived cytokinins systemically regulate root nodulation. *Nat. Commun.* 5 4983 10.1038/ncomms5983 25236855

[B110] Schuller A. Kehr J. Ludwig-Müller J. (2014). Laser microdissection coupled to transcriptional profiling of *Arabidopsis* roots inoculated by *Plasmodiophora brassicae* indicates a role for brassinosteroids in clubroot formation. *Plant Cell Physiol.* 55 392–411. 10.1093/pcp/pct174 24285749

[B111] Schüβler A. Schwarzott D. Walker C. (2001). A new fungal phylum, the Glomeromycota: phylogeny and evolution. *Mycol. Res.* 105 1413–1421. 10.1017/S0953756201005196

[B112] Schweiger R. Müller C. (2015). Leaf metabolome in arbuscular mycorrhizal symbiosis. *Curr. Opin. Plant Biol.* 26 120–126. 10.1016/j.pbi.2015.06.009 26202872

[B113] Sharaf E. F. Farrag A. A. (2004). Induced resistance in tomato plants by IAA against *Fusarium oxysporum* lycopersici. *Pol. J. Microbiol.* 53 111–116. 15478356

[B114] Shaul-Keinan O. Gadkar V. Ginzberg I. Grünzweig J. M. Chet I. Elad Y. (2002). Hormone concentrations in tobacco roots change during arbuscular mycorrhizal colonization with *Glomus intraradices*. *New Phytol.* 154 501–507. 10.1046/j.1469-8137.2002.00388.x 33873426

[B115] Siemens J. Keller I. Sarx J. Kunz S. Schuller A. Nagel W. (2006). Transcriptome analysis of *Arabidopsis* clubroots indicate a key role for cytokinins in disease development. *Mol. Plant Microbe Interact.* 19 480–494. 10.1094/MPMI-19-0480 16673935

[B116] Singh S. Parniske M. (2012). Activation of calcium- and calmodulin-dependent protein kinase (CCaMK), the central regulator of plant root endosymbiosis. *Curr. Opin. Plant Biol.* 15 444–453. 10.1016/j.pbi.2012.04.002 22727503

[B117] Smith E. F. Townsend C. O. (1907). A plant-tumor of bacterial origin. *Science* 25 671–673. 10.1126/science.25.643.671 17746161

[B118] Smith S. E. Read D. J. (2010). *Mycorrhizal Symbiosis.* Cambridge, MA: Academic Press.

[B119] Soltis D. E. Soltis P. S. Morgan D. R. Swensen S. M. Mullin B. C. Dowd J. M. (1995). Chloroplast gene sequence data suggest a single origin of the predisposition for symbiotic nitrogen fixation in angiosperms. *Proc. Natl. Acad. Sci. U.S.A.* 92 2647–2651. 10.1073/pnas.92.7.2647 7708699 PMC42275

[B120] Splivallo R. Fischer U. Göbel C. Feussner I. Karlovsky P. (2009). Truﬄes regulate plant root morphogenesis via the production of auxin and ethylene. *Plant Physiol.* 150 2018–2029. 10.1104/pp.109.141325 19535471 PMC2719122

[B121] Sturtevant D. B. Taller B. J. (1989). Cytokinin production by *Bradyrhizobium japonicum*. *Plant Physiol.* 89 1247–1252. 10.1104/pp.89.4.1247 16666691 PMC1056003

[B122] Suzaki T. Ito M. Kawaguchi M. (2013). Induction of localized auxin response during spontaneous nodule development in *Lotus japonicus*. *Plant Signal. Behav.* 8:e23359 10.4161/psb.23359 PMC3676504 23299335

[B123] Suzaki T. Yano K. Ito M. Umehara Y. Suganuma N. Kawaguchi M. (2012). Positive and negative regulation of cortical cell division during root nodule development in *Lotus japonicus* is accompanied by auxin response. *Development* 139 3997–4006. 10.1242/dev.084079 23048184

[B124] Svistoonoff S. Hocher V. Gherbi H. (2014). Actinorhizal root nodule symbioses: what is signalling telling on the origins of nodulation? *Curr. Opin. Plant Biol.* 20 11–18. 10.1016/j.pbi.2014.03.001 24691197

[B125] Tirichine L. Sandal N. Madsen L. H. Radutoiu S. Albrektsen A. S. Sato S. (2007). A gain-of-function mutation in a cytokinin receptor triggers spontaneous root nodule organogenesis. *Science* 315 104–107. 10.1126/science.1132397 17110537

[B126] Torrey J. G. (1961). Kinetin as trigger for mitosis in mature endomitotic plant cells. *Exp. Cell Res.* 23 281–299. 10.1016/0014-4827(61)90038-6 13777586

[B127] Udvardi M. Poole P. S. (2013). Transport and metabolism in legume-rhizobia symbioses. *Annu. Rev. Plant Biol.* 64 781–805. 10.1146/annurev-arplant-050312-120235 23451778

[B128] van Zeijl A. Op den Camp R. H. M. Deinum E. E. Charnikhova T. Franssen H. Op (2015). *Rhizobium* lipo-chitooligosaccharide signaling triggers accumulation of cytokinins in *Medicago truncatula* roots. *Mol. Plant* 8 1213–1226. 10.1016/j.molp.2015.03.010 25804975

[B129] Wood D. W. Setubal J. C. Kaul R. Monks D. E. Kitajima J. P. Okura V. K. (2001). The genome of the natural genetic engineer *Agrobacterium tumefaciens* C58. *Science* 294 2317–2323. 10.1126/science.1066804 11743193

[B130] Yang J. Kloepper J. W. Ryu C.-M. (2009). Rhizosphere bacteria help plants tolerate abiotic stress. *Trends Plant Sci.* 14 1–4. 10.1016/j.tplants.2008.10.004 19056309

[B131] Yuliar Nion Y. A. Toyota K. (2015). Recent trends in control methods for bacterial wilt diseases caused by *Ralstonia solanacearum*. *Microbes Environ.* 30 1–11. 10.1264/jsme2.ME14144 25762345 PMC4356456

